# Expert Recommendations on the Diagnosis of Eosinophilic Esophagitis in the United Arab Emirates

**DOI:** 10.7759/cureus.56062

**Published:** 2024-03-12

**Authors:** Sameer Al Awadhi, Mohamad Miqdady, Mohamed Abuzakouk, Osama Yousef, Christos Tzivinikos, Filippos Georgopoulos, Stuart Carr, Ahmed Sultan, Rana Bitar, Asad Izziddin Dajani, Mazen Taha, Eyad Alakrad, Ahmad Jazzar, Mohammed Banama, Khaled Bamakhrama, Nawal Alnahdi, Ahmed Ali Elghoudi, Amer Azaz, Ravi Gutta, Monica Fahmy, Boushra Raghib, Suzan Murad, Mina Abdelmallek

**Affiliations:** 1 Gastroenterology, Hepatology, and Endoscopy, Rashid Hospital, Dubai, ARE; 2 Pediatric Gastroenterology, Hepatology, and Nutrition, Sheikh Khalifa Medical City (SKMC), Abu Dhabi, ARE; 3 Pediatric Gastroenterology, Hepatology, and Nutrition, College of Medicine and Health Sciences, Khalifa University, Abu Dhabi, ARE; 4 Allergy and Immunology, Respiratory Institute, Cleveland Clinic, Abu Dhabi, ARE; 5 Gastroenterology, Digestive Disease Institute, Cleveland Clinic, Abu Dhabi, ARE; 6 Pediatric Gastroenterology, Al Jalila Children's Speciality Hospital, Dubai, ARE; 7 Pediatric Gastroenterology, Mohammed Bin Rashid University of Medicine and Health Sciences, Dubai, ARE; 8 Gastroenterology and Hepatology, Al Zahra Hospital, Dubai, ARE; 9 Immunology and Allergy, Snö Clinics, Abu Dhabi, ARE; 10 Gastroenterology and Hepatology, Mediclinic Airport Road Hospital, Abu Dhabi, ARE; 11 Gastroenterology, Dr. Asad Dajani Specialized Clinic, Sharjah, ARE; 12 Gastroenterology and Hepatology, Tawam Hospital, Abu Dhabi, ARE; 13 Gastroenterology and Hepatology, Sheikh Shakhbout Medical City, Abu Dhabi, ARE; 14 Gastroenterology and Hepatology, Burjeel Day Surgery Center, Abu Dhabi, ARE; 15 Pediatric Allergy, Sheikh Khalifa Medical City (SKMC), Abu Dhabi, ARE; 16 Immunology, Mediclinic City Hospital, Dubai, ARE; 17 Medical Department, Sanofi, Dubai, ARE

**Keywords:** gastroesophageal disease, united arab emirates, type 2 inflammation, recommendations and guidelines, eosinophilic esophagitis

## Abstract

Eosinophilic esophagitis (EoE) is a chronic, progressive, type 2 inflammatory esophageal disease presenting as dysphagia to solid food and non-obstructive food impaction. Knowledge gaps exist in its diagnosis and management. These expert recommendations focused on the diagnosis of EoE in the United Arab Emirates.

An electronic search of PubMed and Embase databases was used to gather evidence from systematic reviews, randomized controlled trials, consensus papers, and expert opinions from the last five years on the diagnosis of EoE. The evidence was graded using the Oxford system. Literature search findings were shared with the expert panel. A 5-point scale (strongly agree, agree, neither agree nor disagree, disagree, and strongly disagree) was used, and a concordance rate of >75% among experts indicated agreement. Using a modified Delphi technique, 18 qualified experts provided 17 recommendations.

Eleven statements achieved high agreement, four got moderate agreement, and two got low agreement. Challenges exist in diagnosing EoE, particularly in children. Esophageal biopsies were crucial in diagnosis, irrespective of visible mucosal changes. Further research on diagnostic tools like endoscopic mucosal impedance and biomarkers is needed. Diagnosis relies on esophageal biopsies and symptom-histology correlation; however, tools like EoE assessment questionnaires and endoscopic mucosal impedance could enhance the accuracy and efficiency of EoE diagnosis.

The diagnosis of EoE is challenging since the symptoms seldom correlate with the histological findings. Currently, diagnosis is based on patient symptoms and endoscopic and histological findings. Further research into mucosal impedance tests and the role of biomarkers is needed to facilitate diagnosis.

## Introduction and background

Eosinophilic esophagitis (EoE) is an atopic, chronic, progressive, type 2 inflammatory disorder characterized by dysregulated, non-IgE-mediated type 2 immune response and elevated eosinophils in the esophageal mucosa [1,2]. EoE incidence has notably risen [3], increasing in both pediatric and adult populations in the last 15-20 years [4]. A meta-analysis reported an estimated EoE incidence of 3.7/100,000 individuals per year and a prevalence of 22.7/100,000 individuals [5].

Although it affects all ages, EoE exhibits distinct peaks in pediatric and third-decade populations [6], with higher prevalence among Caucasians and males, linked to atopic conditions, genetics, environmental factors, and fibrous remodeling [4].

The diagnostic criteria includes >15 intraepithelial eosinophils per high-power field in esophageal biopsies [4]. EoE can be diagnosed through symptoms, endoscopic features, and histological findings via esophagogastroduodenoscopy (EGD), with biopsies as the preferred test; it is diagnosed in 2-6.5% of EGD cases [7]. It contributes significantly to dysphagia (12-22% diagnosis rate) and esophageal food impaction (50% diagnosis rate) in adults [7] and can impact children's growth [8]. In younger children, symptoms may be vague [9].

The endoscopic features of EoE commonly include mucosal edema, loss of vascular pattern, linear furrows, white specks, and concentric rings. Multiple biopsies from the proximal and distal esophagus are recommended due to EoE's patchy nature, requiring differentiation from conditions like gastroesophageal reflux disease (GERD) which can also cause esophageal eosinophilia [3]. These expert recommendations aimed to develop United Arab Emirates (UAE)-specific diagnostic criteria for EoE.

The objectives were to understand the gaps in the diagnosis of EoE, the role of biomarkers in the diagnosis of EoE, ways to improve the biopsy procedure, and the link between histology and symptoms of EoE.

## Review

Expert recommendations were framed through the modified Delphi method, comprising an 18-member panel from adult and pediatric gastroenterology and immunology who consented to participate in this consensus. The responses from the experts were collected anonymously. An ethical clearance from the internal review board was not deemed necessary as it was considered to be minimal risk. An electronic search of PubMed and Embase databases was used to gather evidence from systematic reviews, randomized controlled trials, consensus papers, and expert opinions from the last five years on the diagnosis of EoE. The literature search was used to develop prereads that were shared with the expert panel.

The literature was graded as per the Oxford level of evidence. The Oxford level of evidence and the grading system used are explained in Table 1 and Table 2 [10].

**Table 1:** Oxford level of evidence CDR: clinical decision rule; CI: confidence interval; RCT: randomized controlled trial Reference: [10]

Level	1a	1b	1c	2a	2b	2c	3a	3b	4	5
Therapy/prevention, etiology/harm	Systematic review (with homogeneity^b^) of RCTs	Individual RCT (with narrow CI^a^)	All/none^c^	Systematic review with homogeneity^b^ of cohort studies	Individual cohort study (low-quality RCTs, such as <80% follow-up)	Outcomes research, ecological studies	Systematic review (with homogeneity^b^) of case-control studies	Single case-control study	Case series/poor-quality cohort/case-control studies^e^	Expert opinion without clear appraisal/based on first principles, bench research, or physiology
Prognosis	Systematic review (with homogeneity^b^) of inception cohort studies with validated CDR^d^ in different populations	Individual inception cohort study with >80% follow-up with validated CDR^d^ in a single population	All/none case series	Systematic review (with homogeneity^b^) of retrospective cohort studies/untreated control groups in RCTs	Retrospective cohort study/follow-up of untreated control patients in RCT with derived/validated CDR^d^ on split sample^f ^	Outcomes research			Case series, poor-quality prognostic cohort studies^g^	Expert opinion on first principles, bench research, or physiology without clear appraisal/bias
Diagnosis	Systematic review (with homogeneity^b^) of diagnostic studies (level 1); CDR^d^ with multicenter 1b studies	Validating^h^ cohort study with good^i ^reference standards or single-center CDR^4^ testing	Absolute SpPins and SnNouts^j^	Systematic review (with homogeneity^b^) of diagnostic studies with level >2	Exploratory^h^ cohort study with good^i ^reference standards; CDR^d^ after derivation or validated on databases or split sample^f^ only		Systematic review (with homogeneity^b^) of studies with level ≥3b	Non-consecutive study or without consistently applied reference standards	Case-control study, poor or non-independent reference standard	Expert opinion without clear appraisal/based on first principles, bench research, or physiology
Differential diagnosis/symptom prevalence study	Systematic review (with homogeneity^b^) of prospective cohort studies	Prospective cohort study with good follow-up^k^	All or none case series	Systematic review (with homogeneity^b^) of 2b and better studies	Retrospective cohort study or poor follow-up	Ecological studies	Systematic review (with homogeneity^b^) of 3b and better studies	Non-consecutive cohort study or very limited population	Case series or superseded reference standards	Expert opinion without clear appraisal/based on first principles, bench research, or physiology
Economic and decision analyses	Systematic review (with homogeneity^b^) of level 1 economic studies	Analysis based on clinically sensible costs or alternatives; systematic review(s) of the evidence; and including multi-way sensitivity analyses	Absolute better-value or worse-value analyses^l^	Systematic review (with homogeneity^b^) of level >2 economic studies	Analysis based on clinically sensible costs or alternatives; limited review(s) of the evidence or single studies; and including multi-way sensitivity analyses	Audit or outcomes research	Systematic review (with homogeneity^b^) of 3b and better studies	Analysis based on limited alternatives or costs, poor-quality estimates of data, but including sensitivity analyses incorporating clinically sensible variations	Analysis with no sensitivity analysis	Expert opinion without clear appraisal/based on first principles, bench research, or physiology
^a^A minus sign (-) can be used to indicate inconclusive evidence. This comprises a single result with a wide CI or a systematic review with concerning heterogeneity. The evidence is not conclusive, generating only grade D recommendations. Refer to this note for guidance on how to interpret trials/studies with wide CIs. ^b^Homogeneity means no concerning heterogeneity (variations/differences) is present in the results between individual studies. However, not all systematic reviews with statistically significant heterogeneity are necessarily concerning, and not all concerning heterogeneity needs to be significant statistically. Studies concerning heterogeneity should be marked at the end with a "-". ^c^Patients who once died before the treatment became available now survive on it; or when some died before the treatment became available, none now do. ^d^CDR: Algorithm or scoring system used to provide an estimation of the prognosis of a disease or to categorize a diagnosis. ^e^A cohort study of poor quality could have any/all of the following: (a) lack of clear definition for comparison groups; (b) absence of uniform measurement of exposures and outcomes in both exposed and non-exposed individuals, preferably in a blinded and objective manner; (c) failure to identify or adequately control known confounders; and (d) inadequate execution of a thorough and prolonged follow-up of patients. A case-control study of poor quality could have any/all of the following: (a) insufficiently defined comparison groups; (b) failure to measure exposures and outcomes consistently in both cases and controls, preferably in a blinded and objective manner; and (c) neglect in identifying or appropriately controlling known confounders. ^f^Split-sample validation involves dividing a single tranche of information into "derivation" and "validation" samples. ^g^A prognostic cohort study of poor quality can have any of the following: (a) biased sampling in favor of patients who achieved the target outcome; (b) outcome measurement in <80% of study participants; (c) unblinded/non-objective methods of determining outcomes; and (d) non-correction of confounding factors. ^h^Validation studies evaluate the quality of a particular diagnostic test based on existing evidence. Exploratory studies gather data to identify significant factors using regression analysis. ^i^Reference standards that maintain independence from the test and are consistently and objectively administered to all patients are of good standard. Conversely, suboptimal reference standards, while still unbiased regarding the test, may be applied haphazardly. The inclusion of a non-independent reference standard, where the test is integrated into the reference or the testing process impacts the reference, signifies a study of level. ^j^An "absolute SpPin" is a high-specificity diagnostic finding where a positive result rules in the diagnosis. An "absolute SnNout" is a high-sensitivity diagnostic finding where a negative result rules out the diagnosis. ^k^For a differential diagnosis study, a follow-up rate of ≥80% is adequate. Sufficient time should be given for alternative diagnoses to emerge, such as 1-6 months for acute cases and 1-5 years for chronic cases. ^l^Treatments that are better value are either equally effective but less expensive or better and cost the same or are less costly. Treatments that are worse value are either equally effective but more expensive or worse and equally or more costly. Comparison of clinical risks versus benefits of treatments are classified as good, better, bad, or worse										

**Table 2:** Grading system of the Oxford level of evidence *: extrapolations occur when data from one situation is used in another situation that may have important clinical differences compared to the original study Reference: [10]

Code	Recommendation	Quality of evidence	Definition
A	Level 1 consistent studies	High	Further research is very unlikely to change our confidence in the estimate of effect. Several high‐quality studies with consistent results. In special cases, one large, high‐quality multicenter trial
B	Level 2/3 consistent studies or extrapolations* from level 1 studies	Moderate	Further research is likely to have an important impact on our confidence in the estimate of effect and may change the estimate. One high‐quality study. Several studies with some limitations
C	Level 4 studies or extrapolations* from level 2/3 studies	Low	Further research is very likely to have an important impact on our confidence in the estimate of effect and is likely to change the estimate. One or more studies with severe limitations
D	Level 5 studies or any level inconsistent/inconclusive studies	Very low	Any estimate of the effect is very uncertain. Expert opinion. No direct research evidence. One or more studies with very severe limitations

**Figure 1:**
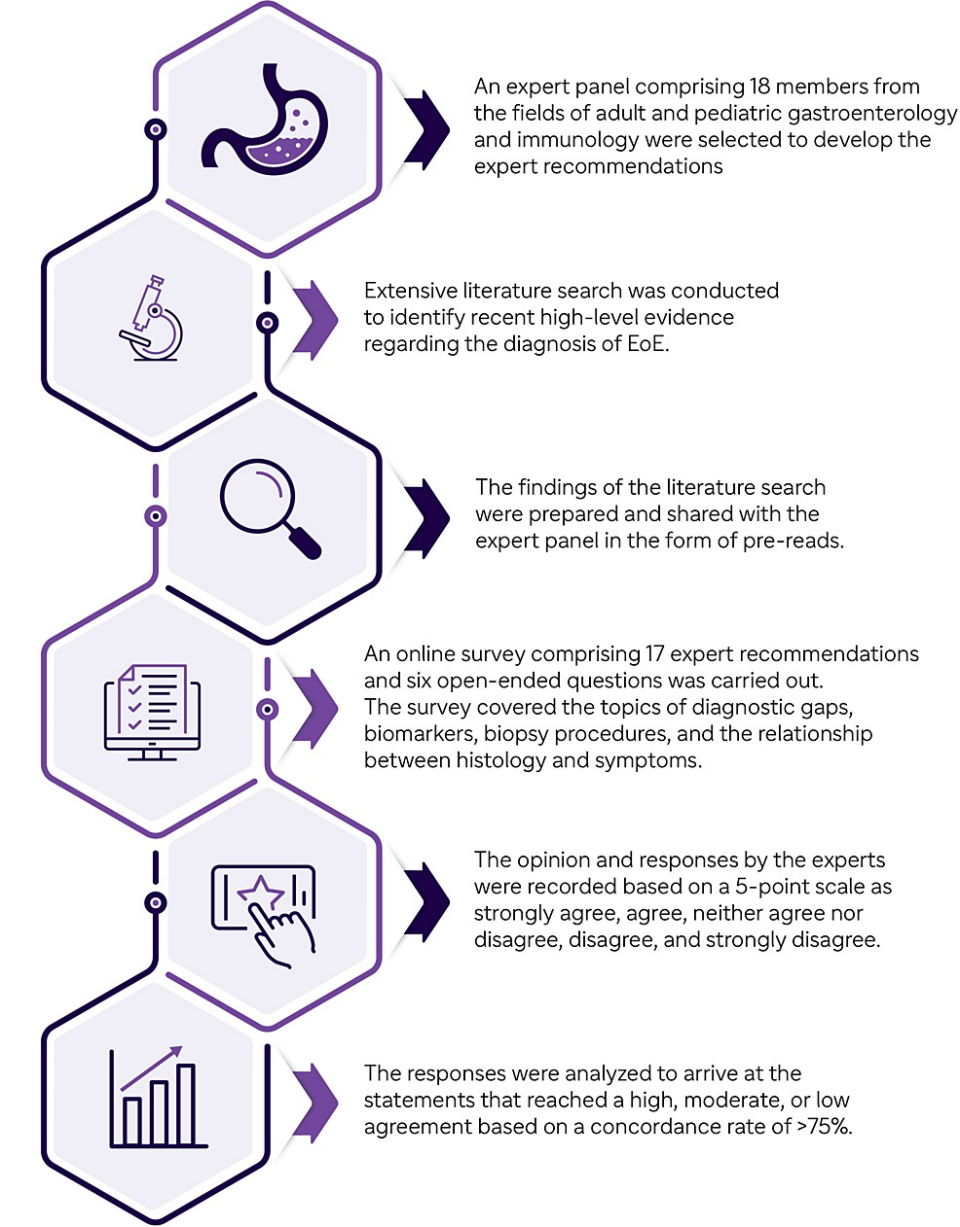
Steps in the development of the expert recommendations EoE: eosinophilic esophagitis Image Credit: All authors

A total of 17 statements were drafted on the gaps in diagnosis, biomarkers, improving procedures for biopsy, and histology-symptom links. An online survey comprising six open-ended and 17 closed-ended questions was shared with the experts. The experts were asked to pick one option from the 5-point scale of strongly agree, agree, neither agree nor disagree, disagree, and strongly agree to answer the 17 close-ended questions. A concordance rate of >75% indicated agreement. The responses were analyzed to categorize statements as having high, moderate, or low agreement. The steps followed to develop the expert recommendations are given in Figure 1.

The criteria to assess agreement levels among the expert panel in diagnosing EoE are depicted in Table 3.

**Table 3:** Criteria for determining levels of agreement among expert panel on EoE diagnosis EoE: eosinophilic esophagitis

Level of agreement	High	Moderate	Low
Definition	When ≥75% of the participants agree or disagree with a statement	When 55–74% of the participants agree or disagree with a statement	When <55% of the participants agree or disagree with a statement

Two rounds of the modified Delphi method were required to finalize 17 clinical statements, which were discussed in the first Delphi round. Along with the clinical statements, six questions on clinical practice were also discussed. Of the 17 clinical statements that underwent online polling, three reached moderate or low consensus. A physical meeting addressed the three clinical statements with moderate or low agreement, and these were reframed. A re-voting determined the final statements.

A total of 17 statements were formulated in the first round of the modified Delphi method, and three of these statements were reframed in the second round (Table 4 and supplementary table (see Appendices), respectively).

**Table 4:** Expert recommendations and their levels of agreement among the expert panel AI: artificial intelligence; EEsAI: eosinophilic esophagitis activity index; EGD: esophagogastroduodenoscopy; ECP: eosinophil cationic protein; EndoFLIP®: endoluminal functional lumen imaging probe; EoE: eosinophilic esophagitis; EUS: endoscopic ultrasound; GERD: gastroesophageal reflux disease; HRM: high-resolution manometry; HPF: high-power field; IL: interleukin; IgE: immunoglobulin E; PPI: proton pump inhibitor; PRO: patient-related outcome

Sl. no.	Expert recommendations	Strongly agree (%)	Agree (%)	Neutral (%)	Disagree (%)	Strongly disagree (%)	Level of agreement	Level of evidence	Grade of evidence
Section 1: Gaps in diagnosis									
1	Esophageal biopsies are necessary for patients with clinical suspicion of EoE, even in the absence of endoscopic alterations of the esophageal mucosa	94	6	0	0	0	High	5	D
2	EoE and GERD can coexist and share a complex relationship with overlapping symptoms; however, EoE exhibits poor response to PPIs and may be associated with a family history of atopic diseases	39	33	11	11	6	Moderate	5	D
3	Patients with EoE present a broad range of variable symptoms leading to underdiagnosis or misdiagnosis and long diagnostic delays	72	22	0	6	0	High	2b	C
4	Mucosal impedance can monitor treatment response in people with EoE by measuring the conductivity across the esophageal epithelium	22	22	44	6	6	Low	5	D
5	Patients with EoE commonly have coexisting atopic conditions, necessitating greater awareness of the disease to facilitate the identification and management of comorbidities	67	33	0	0	0	High	5	D
6	The underlying cause of EoE is unknown; there exists a complex interaction between environmental and genetic factors, the implications of which are yet to be elucidated	61	33	6	0	0	High	5	D
7	Further research is required on PROs in EoE, including the identification and validation of instruments in addition to existing tools	56	39	5	0	0	High	5	D
8	In patients with EoE remission, symptoms should be monitored continuously with periodic endoscopies and biopsies	17	72	0	11	0	High	5	D
9	How often do you monitor symptoms or scope patients in your practice?	The survey responses indicate that variations exist in symptom monitoring and scope examinations in clinical practice. In practice, the symptom monitoring frequency ranges from every two months to once in 8-12 weeks, with adjustments based on treatment stage and patient stability. Respondents suggested monitoring symptoms every 3-6 months or every three months. Opinions on endoscopy intervals also differed, with recommendations for annual, six-monthly, or post-treatment endoscopic examinations. Notably, a few participants disagreed with routine endoscopy if the patient is in remission and symptom-free. Initially, more frequent monitoring was suggested, such as every 3-6 months or monthly. Overall, these findings highlight the need for individualized monitoring plans based on the patient's condition and treatment response							
10	Eosinophil enumeration and evaluation of histology remain the gold standards for EoE monitoring	78	22	0	0	0	High	5	D
11	The use of different (non-EoE-specific or non-validated) instruments to assess EoE symptom severity may explain the dissociation between EoE symptom severity and histologic activity	28	50	17	5	0	High	5	D
12	What instruments do you use or feel are desirable to use to measure EoE activity?	The survey responses highlight a variety of instruments used or considered desirable for use in assessing the disease activity of EoE. These instruments include the EEsAI, validated dysphagia scores, symptom scores, endoscopy, and histological scores. Non-invasive biomarkers and clinical symptom scoring systems are also mentioned as valuable tools. Biopsies, EGD, and dysphagia symptom questionnaires are commonly utilized. Additionally, techniques such as mucosal impedance, EndoFLIP®, string tests, and sponge tests are suggested for comprehensive evaluation. In summary, a combination of symptom assessment, EGD, histology, and specialized questionnaires, including the EEsAI, is commonly used to measure EoE activity in adults and the Pediatric EoE Symptom Score (PEESS® v2.0) in children. The inclusion of non-invasive biomarkers and additional tests further enhances the evaluation process							
13	Although esophageal motility is not routinely assessed in clinical practice, it is a valuable aid in the monitoring and management of EoE	11	33	28	22	6	Low	5	D
14	Diagnostic tools such as EndoFLIP® and EUS with HRM aid in identifying dysphagia and treatment needs in patients with EoE	22	33	45	0	0	Moderate	5	D
15	AI, an innovative, non-invasive diagnostic tool, is useful in predicting the risk of EoE, detecting EoE, and improving preventive care and management of EoE	11	56	33	0	0	Moderate	5	D
16	PPIs are considered as one of the first-line options for the treatment of EoE, irrespective of patient's age, and as an initial treatment option before diagnosis, owing to their safety and cost-effectiveness	33	33	6	28	0	Moderate	2b	C
Section 2: Literature on biomarkers									
17	Several promising minimally invasive biomarkers for EoE have been developed; however, only a few differentiate EoE from other atopic diseases	17	67	11	5	0	High	1a	A
18	Are you aware of any biomarkers with the potential to identify EoE?	The survey responses indicate a mixed level of awareness regarding biomarkers for identifying EoE. While some respondents were aware of potential biomarkers, others expressed their lack of knowledge in this area. Some respondents mentioned this as a topic of ongoing research and the lack of validation of biomarker use for diagnostic purposes. In summary, the survey revealed varying levels of familiarity with EoE biomarkers, with some respondents acknowledging their importance while others highlighting the need for further research and validation							
19	Have you used any biomarkers to identify EoE?	Most of the respondents in the survey stated that they have not used any biomarkers to identify EoE. However, one respondent mentioned the use of ECP as a biomarker, and another respondent mentioned the use of IL25 and IL44 as potential biomarkers for EoE. Additionally, eosinophils and total IgE in atopic patients were mentioned as potential biomarkers for EoE. Overall, the use of biomarkers for identifying EoE appeared to be limited among the respondents, with only a few specific biomarkers being mentioned							
Section 3: Improving procedures for taking biopsies									
20	Multiple biopsies from different sites (upper, middle, and lower thirds of the esophagus) having predominant eosinophilic inflammation in the esophagus are considered optimal for arriving at a diagnosis	72	22	0	0	6	High	5	D
21	How many biopsies do you take during scoping?	The practitioners who took the survey provided varying responses regarding the number of biopsies taken during esophagoscopy for EoE. While some did not specify a specific number, others mentioned taking three, six, or eight. One approach that they suggested was obtaining two biopsies from each third of the esophagus, resulting in a total of six biopsies. The respondents felt that gastroenterologists typically performed upper and mid-esophageal biopsies. The respondents felt that it was important to consider the individual cases and healthcare professionals' preferences when determining the appropriate number of biopsies for EoE diagnosis and monitoring							
22	Is there any situation where you take <6 biopsies?	The survey respondents suggested that while most of the practitioners usually take six or more biopsies during esophagoscopy for EoE, there are instances where fewer biopsies may be taken. These situations include when there is a low suspicion or no suspicion of EoE based on symptoms and endoscopic findings. Some respondents mentioned factors such as their concerns about complications, such as bleeding and perforation, may influence the decision to take fewer biopsies. It is important to consider the specific clinical context and individual patient characteristics when determining the appropriate number of biopsies needed for accurate EoE evaluation							
Section 4: Link between histology and symptoms									
23	The diagnosis depends on the clinical features, endoscopy, and a histologic finding of at least 15 eosinophils/HPF in the esophageal biopsy	83	17	0	0	0	High	5	D

EoE is a chronic type 2 inflammatory disorder, showing significant eosinophil infiltration in the esophageal mucosa. Clinicians' understanding of the condition continues to evolve since it was first described just over two decades ago [2] with a global rise in incidence and prevalence [11]. There are ongoing discussions regarding the diagnostic criteria and therapeutic evaluation methods for detecting EoE as early diagnosis can avert fibrostenotic disease in patients [8].

Discerning EoE from GERD poses challenges due to varying symptoms across age groups and overlapping manifestations [12]. A cohort study highlighted EoE's natural progression from inflammatory to fibrostenotic, emphasizing that each year of undiagnosed EoE elevates the risk of developing strictures by around 9% [11]. Therefore, these expert recommendations aimed to bridge the gap in knowledge regarding the various approaches to the diagnosis of EoE.

Gaps in diagnosis

Diagnosis Based on Clinical Presentation and Biopsies

The panelists unanimously supported esophageal biopsies in diagnosing EoE, even without visible mucosal alterations (level of evidence: 5; grade of evidence: D). This aligns with the recommendation for esophageal biopsies when clinically suspecting EoE, regardless of any visible changes in the esophageal mucosa. Biopsies from multiple sites in different esophageal thirds to detect patchy eosinophilic infiltrates are desirable [13].

The panelists agreed that the accurate and early diagnosis of EoE is challenging due to diverse symptoms (level of evidence: 2b; grade of evidence: C). Adults commonly manifest characteristic symptoms such as dysphagia and food impaction facilitating the diagnosis, while pediatric patients exhibit a broader spectrum of nonspecific symptoms, making diagnosis more challenging and emphasizing the need for vigilance in at-risk patients. Navarro et al. reported that the substantial diagnostic delays encountered can significantly impact patient outcomes and hinder the timely initiation of appropriate interventions [14].

There is limited knowledge of the cytokine-mediated immune activation by the eosinophilic infiltration in EoE and its effects on fibrosis and tissue remodeling of the esophagus. These changes may alter the structural and mechanical properties of the esophagus, contributing to symptoms like dysphagia and food bolus impaction [15]. A recent systematic review reports that dysmotility resolves after medical treatment, suggesting that eosinophilic infiltration and the associated inflammation contribute to esophageal dysfunction in EoE. However, further research is needed to understand the complex interactions underlying esophageal dysmotility in EoE [16].

The expert panel suggested comprehensive management strategies in EoE patients with comorbid atopic conditions (level of evidence: 5; grade of evidence: D). Specific allergy history questions were recommended for enhanced diagnostic accuracy and treatment planning. A significant percentage of EoE patients have asthma or other coexisting conditions underscoring the need for a thorough family and personal medical history. Probing for allergy-related symptoms enables clinicians to facilitate a more accurate diagnosis [17]. However, a meta-analysis failed to establish an accurate association between individuals with atopy and EoE [18]. Allergy tests play a valuable role in assessing coexisting atopic conditions in EoE patients. More recently, a cross-sectional analysis of EoE symptoms using data from the CONNECT registry, which performed skin or serum allergy tests, reported limited effectiveness in identifying the underlying causes of the disease and guiding treatment [14]. Consequently, the American College of Gastroenterology (ACG) 2013 guidelines do not endorse the routine use of these tests, though referrals to allergy clinics for comprehensive evaluations are common [14].

The experts suggested that although the precise etiology of EoE remains incompletely understood, it is postulated to stem from a confluence of genetic and environmental factors (level of evidence: 5; grade of evidence: D). Allergies, alongside other contributory factors such as infections, are considered potential underlying causes when discerning EoE from alternative diagnoses. Carr et al. reported that established risk factors include atopy and genetic/environmental factors such as alterations in gut barrier function, antigen exposure, aeroallergen exposure, early microbial exposure, and fibrous remodeling [4].

The hygiene hypothesis suggests that reduced early-life exposure to microbes in developed countries may contribute to the increasing prevalence of EoE, leading to immune tolerance defects and heightened susceptibility to allergic diseases [19].

The commensal microflora of the gut, through their influence on the Th2 T cells, particularly in cases of early-life exposures like preterm birth, increase the risk of EoE [19]. Cross-sectional analyses reported an inverse relationship between *Helicobacter pylori* exposure and EoE; *H. pylori* equips the immune system against a Th1 environment, while its absence leads to a Th2 immune environment as observed in EoE [20].

Regular endoscopies and biopsies during EoE remission are recommended for monitoring inflammation (level of evidence: 5; grade of evidence: D). The expert panel discussed the significance of symptom scores for understanding EoE symptoms and their potential as a baseline for future research. While endoscopy with biopsy remains the gold standard, caution was advised regarding the subjective nature of the endoscopic reference score, suggesting that additional diagnostic methods are required for diagnosing EoE (level of evidence: 5; grade of evidence: D). Despite the limitations of biopsy like potential bias and variability, the panel strongly agrees on eosinophil enumeration and histology as the gold standard. They attribute the symptom-histology activity discrepancy to the use of non-validated instruments, a consensus reached with a high level of agreement.

Differentiating Between EoE and GERD and the Role of Proton Pump Inhibitors (PPIs) in the Treatment of EoE

It is hypothesized that GERD may trigger EoE by inducing alterations in the integrity of the esophageal mucosa, facilitating the passage of allergens and resulting in an allergic immune response [21].

The expert panel moderately agreed on the potential coexistence of EoE and GERD, noting their complex relationship with overlapping symptoms (level of evidence: 5; grade of evidence: D). EoE may respond poorly to PPIs, but caution was advised, considering the diverse disease phenotypes that could influence individual responses to PPI treatment. The experts recommended evaluating the response to PPI based on the patient's phenotypic characteristics.

This aligns with Carr et al. indicating that the EoE patients have a limited response to acid suppression therapy, such as PPIs, despite symptom overlap with GERD. Additionally, up to 75% of EoE patients have a history of atopic disease, including asthma, eczema, allergic rhinitis, and food allergies [4].

For the statement that PPIs are commonly used as the initial treatment option for EoE, irrespective of age, the expert panel reached a moderate level of agreement (level of evidence: 2b; grade of evidence: C). The experts added scoping before PPI administration in adults; however, in children, PPIs were the preferred first-line treatment option as an empirical food elimination strategy, which is also one of the first-line treatments; however, this can be challenging in children [9].

Role of Assessment Tools

Clinicians assess EoE activity using clinician-reported outcome (ClinRO) measures, evaluating various endoscopic, histologic, functional, and laboratory findings. Besides ClinRO measures, patient-reported outcome (PRO) measures are also utilized which have been validated by the US Food and Drug Administration (FDA). These instruments aim to enhance patient-reported data quality in medical research and regulatory decision-making. PRO measures, self-reported by patients, assess symptom severity, health-related quality of life, general quality of life, or health status [22].

Validated instruments for assessing the severity of EoE symptoms and quality of life are now available. These validated instruments contribute to improved clinical assessments and research in EoE management and patient care [22]. However, the panel emphasized the need for further research to validate the use of these instruments in EoE assessment (level of evidence: 5; grade of evidence: D).

Advanced Diagnostic Tools

Advanced imaging, like optical coherence tomography (OCT), and artificial intelligence (AI) and convolutional neural networks (CNNs) to analyze OCT images [23] have provided high-resolution images to identify and measure the five layers of esophageal tissue, particularly for detecting thickening of the basal layers, which is a characteristic of EoE, allowing its diagnosis with 97% accuracy [2].

The functional luminal imaging probe (FLIP), an FDA-approved measurement tool (EndoFLIP®), complements the traditional assessment of the esophagus. It simultaneously measures the pressure, diameter, and mechanical properties of the esophageal wall and the dynamics of the esophagogastric junction in different esophageal diseases. The EndoFLIP® employs high-resolution impedance planimetry while performing volume-controlled distention to measure the luminal cross-sectional area (CSA) along an axial plane [24].

High-resolution manometry (HRM) is used to evaluate esophageal motor function via esophageal pressure topography (EPT). The HRM/EPT technology provides a comprehensive identification of distinct clinical phenotypes of esophageal motor disorders and a detailed assessment of esophageal function, aiding the diagnosis and management of various esophageal conditions [25].

Diagnostic tools such as endoscopic FLIP and endoscopic ultrasound (EUS) with HRM assist in identifying dysphagia and treatment needs. There was a moderate level of agreement among the expert panel regarding this as EUS may not be feasible in all cases of EoE (level of evidence: 5; grade of evidence: D).

The experts, therefore, suggested splitting the original statement (Table 2) into two statements (Table 3): (1) Diagnosis in patients with EoE may be further aided by tools such as EndoFLIP® and EUS with HRM; however, their use is limited due to lack of standardization and routine availability in typical clinical practice. (2) The cost of EndoFLIP® and EUS with HRM may pose a challenge to their routine use in identifying dysphagia and treatment needs.

The expert panel moderately agreed that AI shows promise in predicting EoE risk, detecting the condition, and enhancing preventive care (level of evidence: 5; grade of evidence: D). They emphasized AI's potential as a complementary tool in EoE diagnosis and management, providing accurate analysis of eosinophil counts, biomarkers, and endoscopic features in the future.

Ambulatory multichannel intraluminal impedance-pH (MII-pH) monitoring is the gold standard for assessing esophageal content. It evaluates reflux episodes and other esophageal events by recording electrical impedance [26].

Lei et al. reported that endoscopic mucosal impedance measurement enables the immediate differentiation of esophageal disorders and can guide treatment decisions by predicting therapeutic responses [26]. However, the experts' opinions varied on the use of mucosal impedance to monitor treatment response in EoE patients due to issues with the availability and clinical expertise required for this technique. The panel disagreed on routine esophageal motility assessment in clinical practice and remained inconclusive about its diagnostic use (level of evidence: 5; grade of evidence: D).

Swallowed and retrievable devices such as the string test (which is a 90-cm encapsulated string) and the Cytosponge (ingestible gelatin capsules consisting of a mesh attached to a string) [21] can capture luminal eosinophil-derived proteins, absolute eosinophil count (AEC), and eosinophil progenitor cells. However, their sensitivity and specificity for diagnosing EoE are inferior to the EoE endoscopic reference score [2,27].

Literature on biomarkers

Multiple studies have investigated non-invasive diagnostic methods for EoE, but only a few biomarkers (serum eosinophil count) exhibit a correlation with disease activity [21]. The identification of a reliable, non-invasive, or minimally invasive biomarker for diagnosing and monitoring EoE could significantly reduce the necessity for invasive procedures, potentially enhancing safety and reducing healthcare costs. A systematic review reported that blood-based biomarkers such as peripheral blood AEC, eosinophilic cationic protein (ECP), eosinophil-derived neurotoxin (EDN), eosinophil peroxidase (EPX), and major basic protein (MBP) play a crucial role in the eosinophil activity and inflammation associated with EoE. However, a consensus paper published in 2017 stated that biomarkers are of poor diagnostic accuracy and are not necessarily elevated in the serum of EoE patients [21]. Various drawbacks such as the timing of specimen collection, patient selection, and the inclusion of an atopic control group have now paved the way for newer non-invasive methods, such as the esophageal string test and Cytosponge [21,27].

The expert panel felt that several minimally invasive biomarkers hold promise in diagnosing EoE, but only a few demonstrate effective discriminatory capabilities between EoE and other atopic diseases (level of evidence: 1a; grade of evidence: A). They emphasized the transformative potential of a reliable and accurate biomarker for EoE, eliminating the need for invasive diagnostic techniques. Hines et al. identified promising minimally invasive biomarkers for EoE; however, these have a limited capacity to differentiate EoE from other atopic diseases [27].

Improving procedures for biopsies

The intraepithelial eosinophils in esophageal biopsies remain the gold standard for the diagnosis of EoE. Pathologists have devised an EoE histology scoring system (HSS) to assess tissue findings in EoE, such as fibroblasts in the lamina propria, which helps to quantify the severity of EoE based on histological observations [28].

Multiple biopsies from different esophageal sites (upper, middle, and lower thirds) are highly recommended for an accurate EoE diagnosis; the expert panel reached a high level of agreement on this (level of evidence: 5; grade of evidence: D).

The joint consensus guidelines by the British Society of Gastroenterology (BSG) and the British Society of Paediatric Gastroenterology, Hepatology, and Nutrition provide evidence-based recommendations for diagnosing and managing EoE in both children and adults. The European Society for Paediatric Gastroenterology, Hepatology, and Nutrition (ESPGHAN) recent guidelines recommend esophageal biopsies in adults undergoing endoscopy if they exhibit endoscopic signs or symptoms associated with EoE, even without visible abnormalities [9]. In children with upper gastrointestinal and PPI-resistant GERD symptoms, endoscopy and biopsy are suggested to exclude EoE. Routine endoscopy and biopsies for adults with typical GERD symptoms refractory to PPI are generally not advised unless specific clinical features suggestive of EoE are present. Esophageal biopsies are recommended during the initial endoscopy for patients with food bolus obstruction to aid in the diagnosis of EoE. A minimum of six biopsies from different anatomical sites (particularly from visible lesions) within the esophagus must be obtained for initial diagnosis and follow-up evaluation of EoE [29].

Link between histology and symptoms

Endoscopy with esophageal biopsy remains the gold standard and the most reliable diagnostic test for EoE. For an accurate diagnosis of EoE, clinicians must assess the patient's symptoms, clinical features, and the extent of eosinophilic infiltration, ruling out other potential causes of esophageal eosinophilia [21].

As per the BSG consensus guidelines, 26% of esophageal biopsies in patients with EoE had food bolus obstruction, while non-obstructive dysphagia was reported in 22% of EoE cases. Although less frequent, reflux and chest pain occur in 6% of EoE patients and should be considered when diagnosing the condition [29].

The expert panel reached a high level of agreement and highlighted the importance of clinical features, endoscopy, and histologic findings (≥15 eosinophils/HPF) for EoE diagnosis (level of evidence: 5; grade of evidence: D). They cautioned against overdiagnosis based solely on eosinophil counts and emphasized evaluating additional histologic features such as inflammation and basal cell hyperplasia. The use of the bite-on-bite biopsy technique was recommended to capture deeper esophageal layers, enhancing diagnostic accuracy.

According to Carr et al., it was observed that the diagnosis of EoE relies heavily on clinical manifestations in patients, endoscopic evaluation of the esophagus, and histologic analysis of esophageal mucosal biopsies [4].

The expert panel agreed that combining various diagnostic techniques, utilizing biomarkers, and leveraging AI were crucial in bridging the knowledge gap in EoE diagnosis. These approaches offer the potential to enhance understanding, improve accuracy, and facilitate more effective management of EoE, ultimately leading to better patient outcomes.

One of the drawbacks of this study was that it utilized a consensus approach, leading to subjective results that were based on expert opinions. The experts' individual experiences and biases could potentially impact the consensus process, which raises concerns about the generalizability of the findings and their ability to comprehensively represent the full range of clinical practice. More research is necessary to corroborate these outcomes.

## Conclusions

Diagnosing EoE presents challenges, particularly in children with nonspecific symptoms. Esophageal biopsies remain vital for diagnosis, regardless of visible mucosal changes. Comprehensive management should consider comorbid atopic conditions, assess allergies, and include a thorough family medical history. Further research is needed to validate PROs and assessment instruments in EoE. Diagnostic tools like endoscopic minimally invasive technique (MIT) and biomarkers show promise for improving EoE diagnosis. This expert recommendation stresses the importance of multiple esophageal biopsies and correlations between histology and symptoms for accurate diagnosis. Ongoing research-enhanced diagnostic procedures and a patient-centered approach are essential for effectively managing and improving patient outcomes.

## Appendices

**Table 5:** Revised expert recommendations EoE: eosinophilic esophagitis; EndoFLIP: endoluminal functional lumen imaging probe; EUS: endoscopic ultrasound; GERD: gastroesophageal reflux disease; HRM: high-resolution manometry; PPI: proton pump inhibitor

Statement number	Original statement	Reframed statement
2	EoE and GERD can coexist and share a complex relationship with overlapping symptoms; however, EoE exhibits poor response to PPIs and has a family history of atopic diseases	Statement 2 has been revised to the following: EoE and GERD can coexist and share a complex relationship with overlapping symptoms; however, EoE is typically associated with a positive personal or family history of atopic disorders and typically has a poorer response to PPIs as compared to GERD, after accounting for phenotypical variations influencing response
14	Diagnostic tools, such as EndoFLIP® and EUS, with HRM aid in identifying dysphagia and treatment needs in patients with EoE	Statement 14 has been revised to the following two statements: Diagnosis in patients with EoE may be further aided by tools such as EndoFLIP® and EUS with HRM; however, their use is limited because of a lack of standardization and routine availability in typical clinical practice. The cost of EndoFLIP® and EUS with HRM may pose a challenge to their routine use in identifying dysphagia and treatment needs in patients with EoE
16	PPIs are considered the first-line option for the treatment of EoE, irrespective of patient's age, and as an initial treatment option before diagnosis, owing to their safety and cost-effectiveness	Statement 16 has been revised to the following: PPIs are considered as one of the first-line options for the treatment of EoE in pediatric patients, whereas, in adult patients, PPIs could be one of the options for treatment, after confirming the diagnosis of EoE through standard endoscopy with esophageal biopsies
